# Ankle and Distal Tibia Megaprostheses in Orthopedic Oncology: A Report of Two Cases and Review of the Literature

**DOI:** 10.15388/Amed.2024.31.2.13

**Published:** 2024-12-04

**Authors:** Edoardo Ipponi, Martina Cordoni, Elena Bechini, Fabrizia Gentili, Fabio Cosseddu, Antonio D’Arienzo, Lorenzo Andreani

**Affiliations:** 1 University of Pisa, Department of Orthopedics and Trauma Surgery, Italy

**Keywords:** Spindle cell sarcoma, Adamantinoma, 3D printed, Custom made prosthesis, Additive manufacturing, Raktažodžiai: Verpstės ląstelių sarkoma, adamantinoma, 3D spausdinimas, pagal užsakymą pagamintas protezas, gamyba

## Abstract

**Background:**

The distal tibia is one of the rarest sites for the onset of malignant and locally aggressive bone tumors. When diagnosed, these lesions should undergo surgical resection with wide margins to eradicate the disease. In the era of limb-sparing surgery, several reconstructive approaches have been proposed and described in modern literature for reconstructing the distal tibia and the ankle. The 3D-printed custom-made prostheses represent an innovative and promising reconstructive option. Several authors highlighted that despite being expensive and prone to some complications such as talar collapse or loosening and soft tissue necrosis, the megaprostheses of the distal tibia could lead to good functional outcomes, also allowing a better range of motion compared to the most common arthrodesis.

**Materials and methods:**

We report two cases who suffered from malignant bone tumors localized in the distal tibia and treated with wide resections and the implant of 3D printed custom-made megaprostheses to replace the distal tibia and the ankle.

**Results:**

Both patients had excellent functional results (MSTS 30/30) one year after surgery. No local recurrence occurred during the patients’ latest follow-up.

**Conclusions:**

Our results support the effectiveness of custom-made implants in replacing the distal tibia and the ankle in orthopedic oncology.

## Abstract (it)

**Įvadas:**

Distalinis blauzdikaulis yra viena iš rečiausių piktybinių ir lokaliai agresyvių kaulų navikų atsiradimo vietų. Diagnozavus šiuos pakitimus, siekiant išgydyti, reikia atlikti plačią chirurginę rezekciją. Galūnes tausojančios chirurgijos eroje šiuolaikinėje literatūroje buvo pasiūlyta ir aprašyta keletas rekonstrukcinių metodų distaliniam blauzdikauliui ir čiurnai rekonstruoti. 3D spausdintuvu pagal užsakymą pagaminti protezai yra novatoriška ir daug žadanti rekonstrukcijos galimybė. Keli autoriai pabrėžė, kad, nepaisant to, jog distalinio blauzdikaulio megaprotezai yra brangūs ir juos naudojant galimos komplikacijos, tokios kaip šokikaulio griuvimas ar atsipalaidavimas ir minkštųjų audinių nekrozė, jie gali lemti gerus funkcinius rezultatus, taip pat suteikti geresnę judesių amplitudę, palyginti su dažniausiai pasitaikančiomis artrodezėmis.

**Metodai:**

Aprašome du atvejus, kai distalinėje blauzdikaulio dalyje buvo lokalizuoti piktybiniai kaulų navikai, kurie buvo gydomi plačiomis rezekcijomis ir implantuojant 3D spausdintuvu pagal užsakymą pagamintus megaprotezus, pakeičiančius distalinę blauzdikaulio dalį ir čiurną.

**Rezultatai:**

Praėjus metams po operacijos abiejų pacientų funkciniai rezultatai buvo puikūs (MSTS 30/30). Per paskutinį pacientų stebėjimą lokalių recidyvų nepasitaikė.

**Išvados:**

Mūsų rezultatai patvirtina, kad pagal užsakymą pagaminti implantai yra veiksmingi pakeičiant distalinį blauzdikaulį ir čiurną ortopedinėje onkologijoje.

## Introduction

The distal tibia is one of the rarest sites for the onset of malignant and locally aggressive bone tumors [1]. When diagnosed, these lesions should undergo surgical resection with wide margins to eradicate the disease [1]. For decades, in the absence of reliable reconstructive options for bone and articulations, below-knee amputation has been the standard treatment for malignant bone tumors arising from the distal tibia. However, amputation is associated with significant psychological, physical, social and financial costs to patients. It was not until the last decades of the last century that the development of more effective chemotherapy drugs and the introduction of modern surgical techniques allowed the spread of modern limb-sparing surgery, representing the current mainstay of treatment for these tumors [2].

After a surgical resection with wide margins, crucial to minimize the risk of local recurrence, orthopedic surgeons are called to reconstruct the distal tibial shaft and the tibial pilon and, when necessary, restore the ankle articulation as a whole [1, 2]. To this date, several options have been described for reconstructing the distal tibia, including endoprosthetic implants, massive osteoarticular allografts, and vascularized autologous grafts. Although arthrodesis are more acceptable by patients, limb function may be severely affected given the loss of ankle motion [3].

The latest technologies in additive manufacturing now allow the production of modern custom-made megaprosthetic implants. Based on the anatomy of the single patient, these prostheses can restore the length and shape of the resected distal tibia and reproduce the function of the ankle articulation [4].

In this study, we report our experience with custom-made distal tibia and ankle megaprostheses and provide an overview of present international literature on the topic.

## Case description

We retrospectively reviewed all the cases with malignant bone tumors of the bone localized in the distal metaphysis or epiphysis of the tibia treated – in the period from 2016 to 2023 – with bone resection and reconstruction using distal femur and ankle megaprostheses.

We identified 45 cases of malignant aggressive bone tumors localized in the tibia. Only two of them were localized in the distal end of the bone near the tibio-talar articulation and required the reconstruction by custom-made megaprosthetic implants (Waldemar Link Gmb & Co. KG, Hamburg, Germany).

For each patient, we collected data regarding their age and gender, as well as previous pathological anamnesis. We recorded the site and size for each tumor, which was estimated using preoperative magnetic resonance imaging and computed tomography scan images. The same images were used to design the custom-made megaprostheses and their relative patient-specific instrumentations (PSIs).

Both cases were treated with one-stage procedures. The resected distal tibia was histopathologically analyzed to confirm the preoperative diagnosis and assess the resection margins.

Postoperative follow-up consisted of serial office visits, clinical evaluations, X-rays, and MRIs. Each complication with a grade II or higher, according to the Clavien–Dindo classification, was recorded. Cases of local recurrence were also reported.

These reports have been performed by the ethical standards of the 1964 Declaration of Helsinki and its later amendments. Patients gave their consent to the treatment and to the drafting of the article.

## Case 1

**Figure 1:**
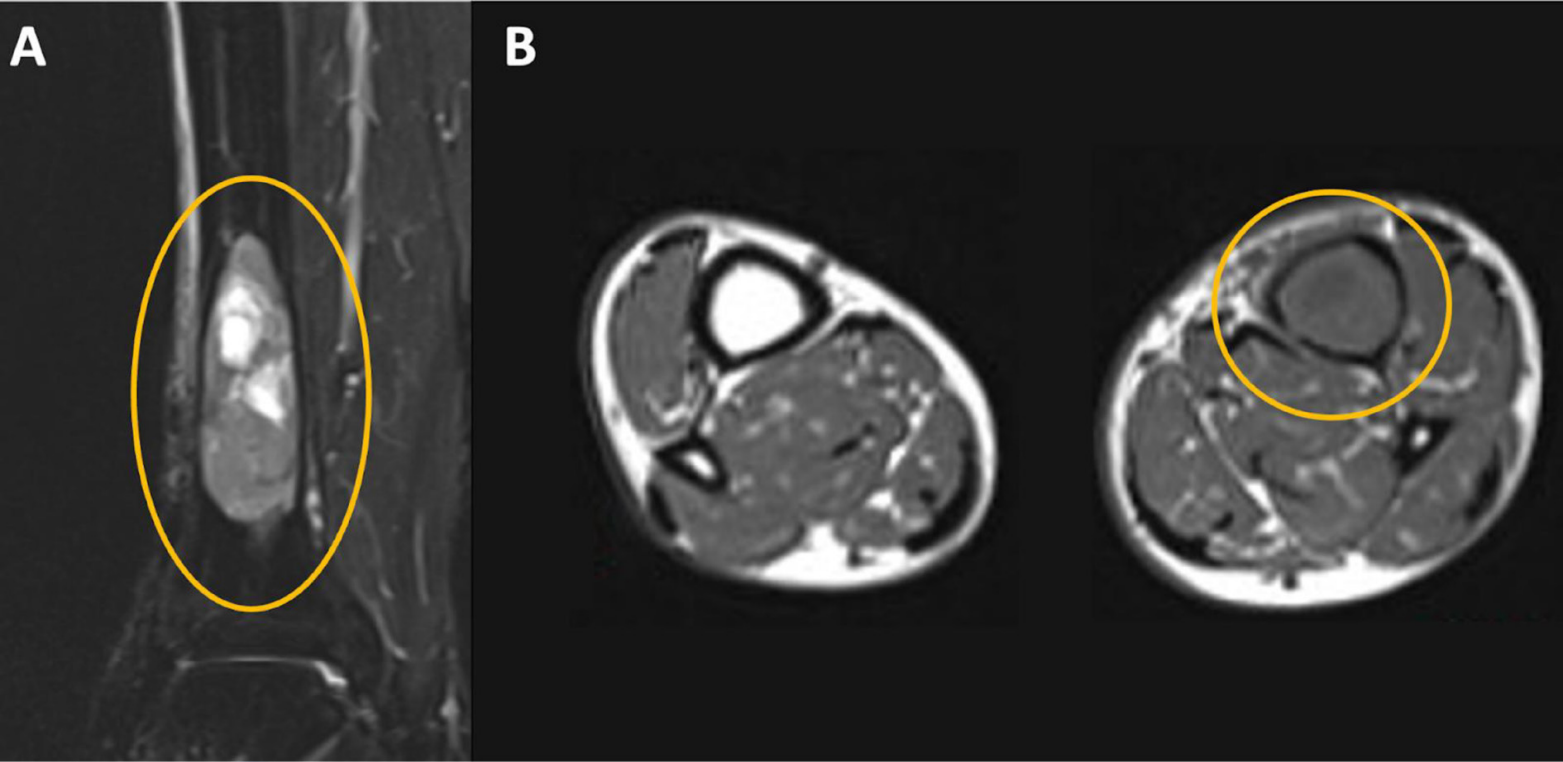
MRI images of Case 1’s neoplasm localized in the distal tibia, viewed on coronal (A) and transverse (B) axes.

A 69-year-old male came to our attention due to persistent pain in his left ankle, which had increased in frequency and magnitude throughout the previous months. Although the foot and ankle did not show swelling or skin alterations, the pain was associated with functional limitations that impaired the patient’s daily activities. His preoperative functional score was 18/30 according to the MSTS scoring scale. X-ray, CT scans, and MRI images revealed a 6-centimeter-wide osteolytic lesion localized in the distal tibia, with signs of cortical scalloping (Figure 1).

An FDG-PET/CT exam highlighted an increased metabolic activity, with a SUVMax of 13.3.

A CT-guided needle biopsy established a histological diagnosis of high-grade spindle cell sarcoma. Based on the preoperative images (CT et al.), Waldemar Ling GmbH & Co., Hamburg, Germany, designed a 3D-printed custom-made distal tibia and ankle prosthesis.

**Figure 2:**
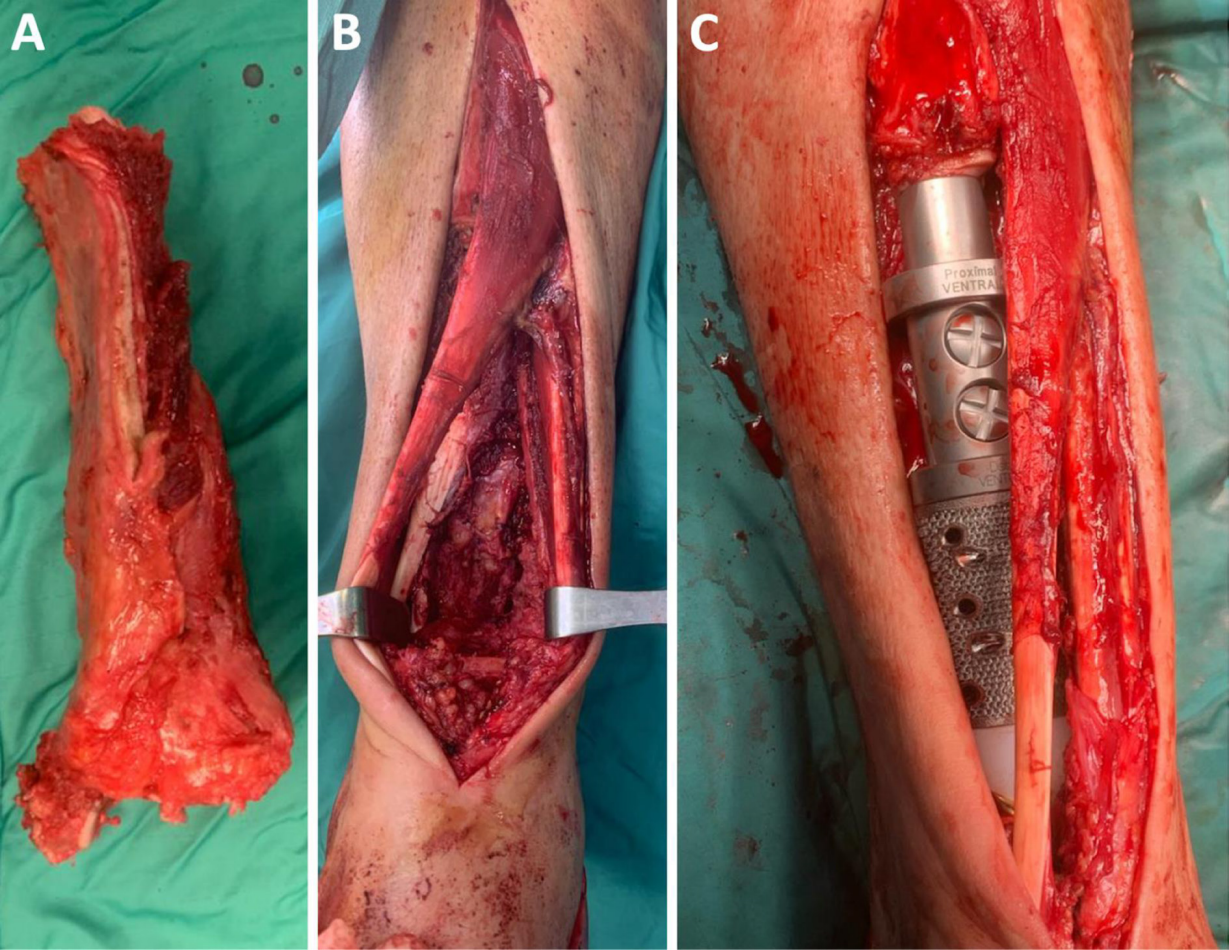
Intraoperative images showing (A) the distal tibia surgical specimen after resection and (B) the resulting bone gap, which was later fulfilled with (C) our custom made prosthetic implant.

In the operative theater, the patient was set on a radiolucent surgical table in a supine position. We performed an anterior approach to the ankle, the tibial pilon, and the distal shaft of the tibia passing between the extensor digitorum longus and the extensor hallucis longus. Proceeding from the anterior end of the bone to its medial and lateral surfaces, we isolated the distal tibia from the nearby muscles, tendons, and connective tissues. The main vascular axes and the major nervous structures were progressively identified and protected through the procedure. Following our preoperative planning, the tibial shaft was cut 125 mm away from the tibio-talar joint line using custom-made cutting jigs. The isolation of the distal tibia was then completed by progressively detaching the flexor digitorum longus, the tibialis posterior, and the flexor hallucis longus from the posterior end of the bone and finally disarticulating the distal tibio-fibular joint and the tibio-tarsal joint. The distal tibia was later delivered to our Pathological Anatomy division, which confirmed the histological diagnosis of spindle cell sarcoma and testified that the resection had been carried out with wide margins. The bone defect was fulfilled with a 3D-printed custom-made distal tibia prosthesis consisting of a Ti6AL4V alloy with a TrabecuLink structure on its bone contact surfaces (Waldemar Link GmbH & Co., Hamburg, Germany), which was fixed to the native tibia with an uncemented stem (Figure 2).

**Figure 3:**
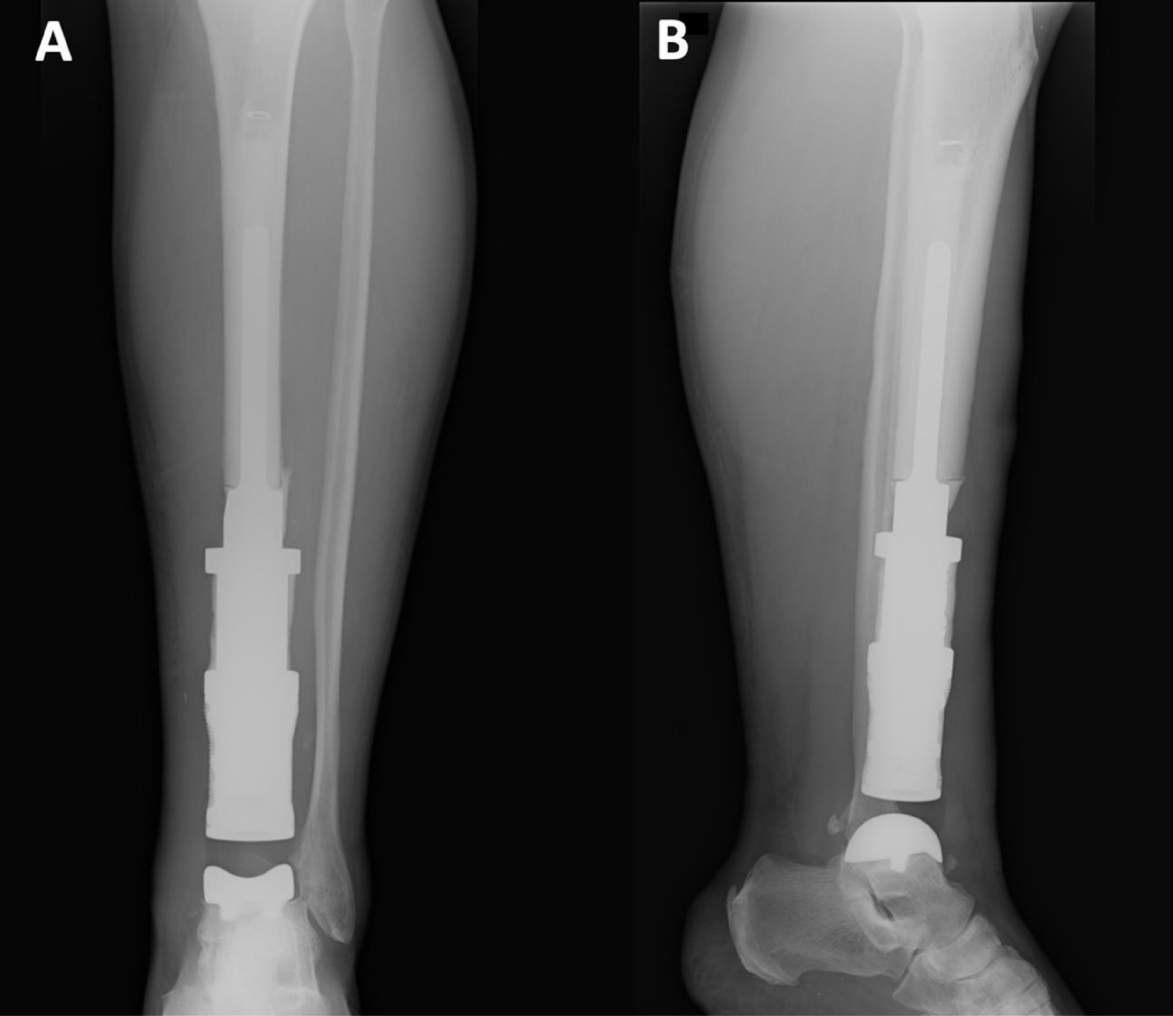
X-rays of the Case 1’s distal tibia and ankle megaprosthesis in an (A) anteroposterior and (B) lateral view.

The tibio-talar articulation was completed with an unconstrained setting by resurfacing the talar dome with a press-fit arthroprosthesis. The articularity of the ankle joint was evaluated before the end of the procedure, intra-operative X-rays were taken (Figure 3), and the surgical access was sutured.

No intra-operative complication occurred. At the end of the intervention, a leg cast was applied and left in place for the following two weeks. Two weeks after surgery, the cast was removed and replaced with a walker boot in a neutral position, which was used part-time for the following two weeks, and the patient was allowed to start active and assisted passive mobilization of his ankle. Within one month after surgery, the patient was allowed to start walking with progressive partial weight bearing on the treated limb, reaching the full weight bearing within eight weeks.

One year after surgery, our clinical evaluations and imaging evidence did not provide evidence of postoperative complications. No local recurrence occurred, and the patient was continuously disease-free (CDF) at his latest follow-up. The patient can now walk without limping and does not require any support. His postoperative MSTS score was 30/30.

## Case 2

**Figure 4:**
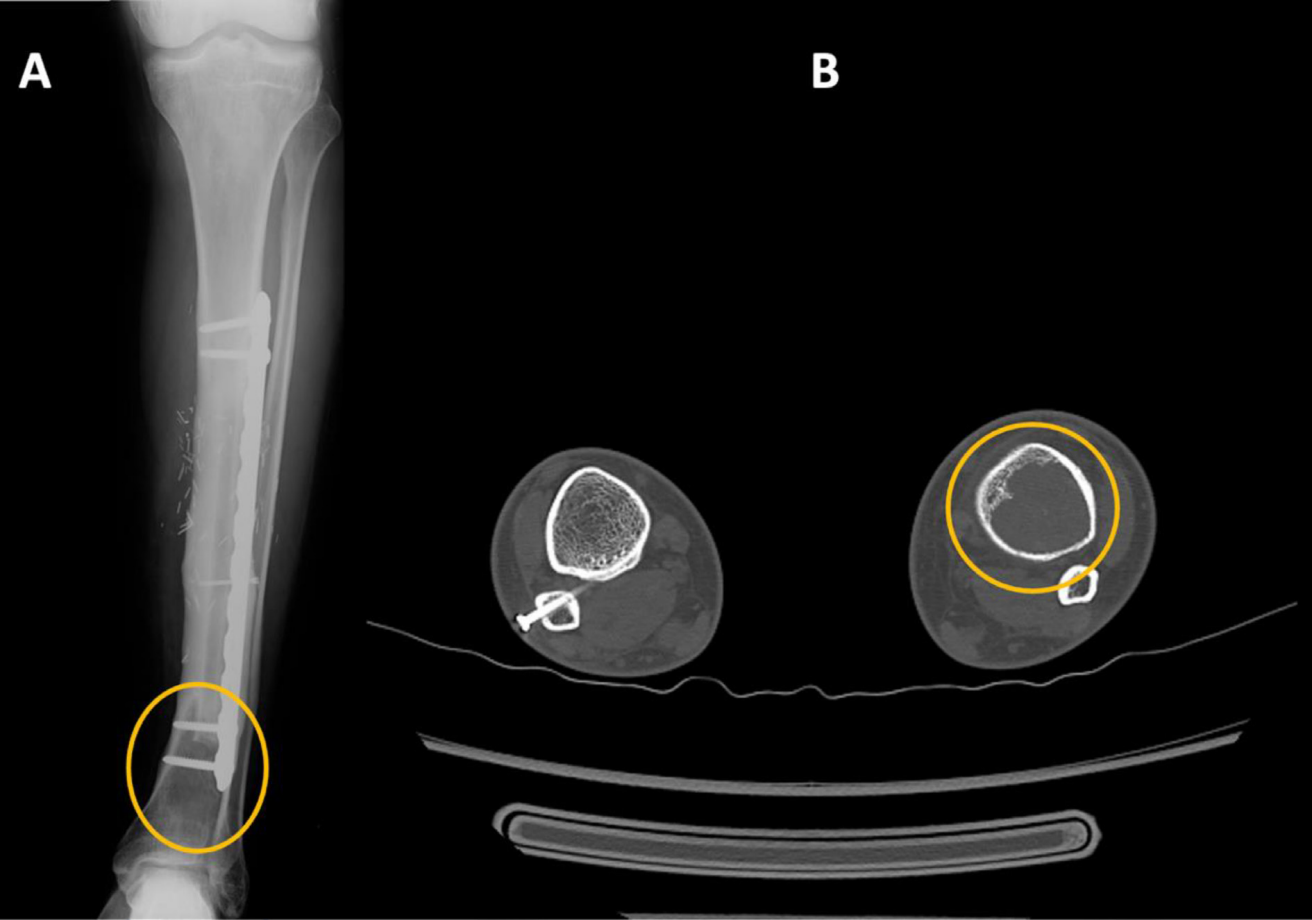
X-ray (A) and CT evidence (B) of an osteolytic lesion localized in the left distal tibia.

Our second case is a 43-year-old male with a history of adamantinoma in his left tibial shaft. Three years before, he had been treated with a vascularized fibular autograft associated with a mio-cutaneous cover from the contralateral leg. After three years of well-being without complications, the routine follow-up with X-rays, MRI, and FDG-PET/TC exams highlighted an osteolytic lesion localized in the left distal tibia, distal to the fibular graft (Figure 4).

A CT-guided needle biopsy confirmed the suspicion of a local recurrence of adamantinoma.

**Figure 5:**
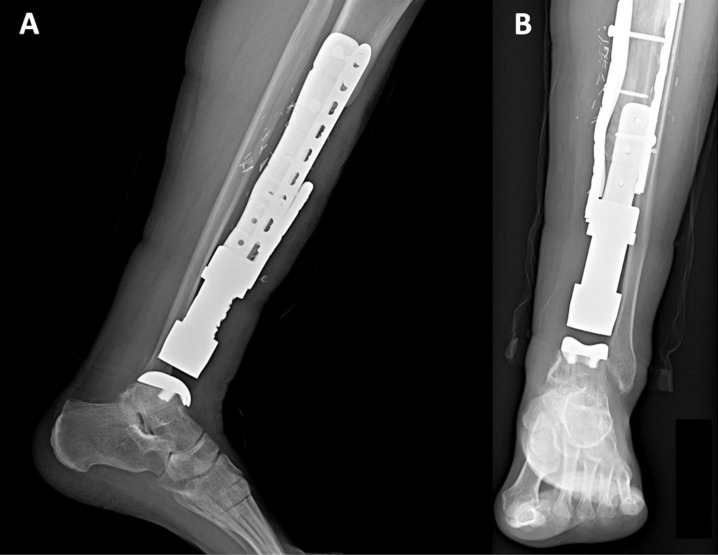
X-rays of the Case 2’s distal tibia and ankle megaprosthesis in a (A) lateral and (B) anteroposterior view.

In consideration of the proximity with the ankle joint and history of our patient, whose native mid-tibial shaft had been replaced by a fibular graft that could not lodge a large intramedullary stem, we opted for a custom-made distal tibia megaprosthesis with an external stabilization, provided by two custom made plates shaped after the unique anatomy of the patient’s bone. An anterior approach to the distal leg and ankle was performed, following the scar lines of the previous intervention. Once we reached the bone, we isolated the distal tibia and disarticulated the ankle joint. The distal autograft bone and the distal plate already in place were then cut according to the preoperative planning, and the distal 10 centimeters of the left tibial bone were removed, achieving wide resection margins. The talar dome was also resected. The bone defect was fulfilled with a 3D-printed custom-made distal tibia prosthesis consisting of a Ti6AL4V alloy with a TrabecuLink structure on its bone contact surfaces (Waldemar Link GmbH & Co., Hamburg, Germany), which was fixed to the remaining autograft and native tibia using custom made medial and anterior plates designed to avoid screw colliding in the tibial shaft. The tibio-talar articulation was completed with a press-fit arthroprosthesis. The articularity of the ankle joint was evaluated, and intra-operative X-rays were taken before the end of the procedure (Figure 5).

**Figure 6:**
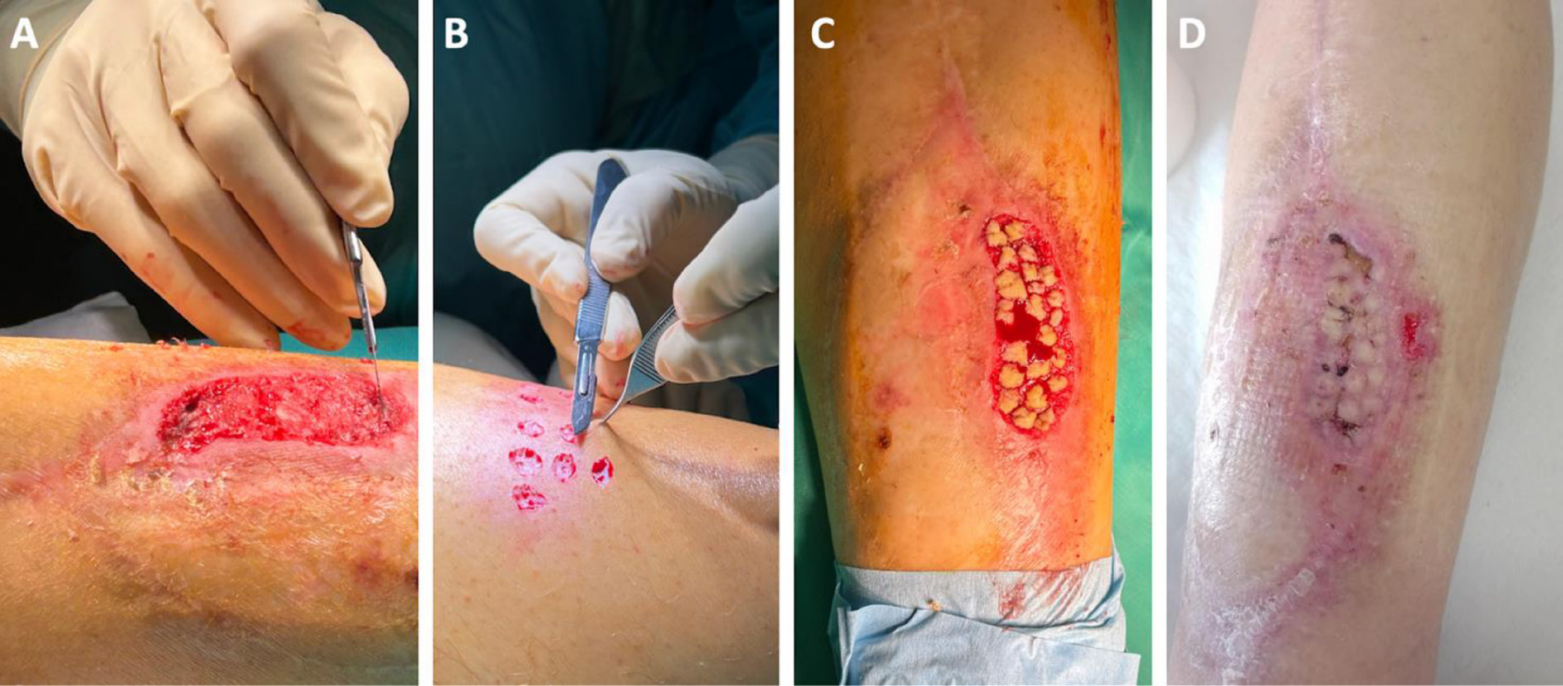
Reverdin skin graft procedure. After (A) a careful debridement of the necrotic area, (B) millimetric skin patches were harvested from the ipsilateral thigh, and (C) used to cover the injured area. A good grade of skin healing could already be seen within three weeks after the procedure (D).

No intra-operative complications occurred. However, a few weeks after surgery, the patient suffered from localized skin necrosis in an area that belonged to the previous myocutaneous graft placed in the previous surgery and was involved in our latest surgical approach. The necrosis was successfully treated with local debridement and a Reverdin skin graft (Figure 6).

Case 2 followed the same postoperative rehabilitation protocol described in Case 1. One year after surgery, our clinical evaluations and imaging evidence did not provide evidence of local recurrence (No Evidence of Disease; NED). The patient can now walk without limping or external support. His postoperative MSTS score was 30/30.

## Discussion

In orthopedic oncology, there has yet to be a consensus on a gold standard technique to replace the distal tibia and the tibiotarsal joint [2]. For decades, arthrodesis has represented the most reliable and used approach for limb-sparing surgery in tumors of the distal leg and ankle region, providing excellent stability and avoiding problems relating to prosthetic implantation [3]. Several authors reported good functional outcomes, with MSTS scores as high as 75–80% and acceptable failure rates in patients with tumors of the distal tibia treated by resection with free vascularized or nonvascularized autologous bone grafts and arthrodesis [3]. However, this surgical procedure has some limitations and can be prone to complications such as nonunion. Arthrodesis implies a total or subtotal limitation of mobility and is often time-consuming, exposing to significant blood losses and theoretically increasing the risk of postoperative infections [3]. Gradually introduced in surgical practice since the dawn of the new millennium, modern endoprosthetic megaprostheses of the distal tibia have been conceived in order to provide an alternative to arthrodeses by minimizing postoperative immobilization, allowing a relatively early recovery and restoring the normal mobility and functionality of the ankle [5-14].

A summary of recent literature on the topic is provided in Table 1.

**Table 1:** **A summary of international literature on distal tibia and ankle megaprostheses**. A systematic review of the literature was carried out in order to identify international literature studies on the distal tibia and ankle megaprostheses in orthopedic oncology. Our analysis was performed by searching in PubMed, Scopus, and Google Scholar the terms “Tibia,” “Ankle,” “Megaprosthesis,” and “Endoprosthesis,” as well as “Sarcoma,” “Bone tumor,” and “Bone metastasis” with all their possible combinations. The research was extended to the studies published in the period from 1985 to December 31st, 2023. The full text of each article potentially suitable for our analysis was viewed independently by two authors (EI and LA). For each study, we recorded the number of cases treated with distal tibia megaprostheses, their mean age, the quality of resection margins, the incidence and type of postoperative complications, the local recurrence rate, patients’ mean MSTS scores and the length of their follow-up.

Study	Year	Cases (N)	Age (y)	Margins	Cases w. Complic.	Infections	Other	L.R. (N)	MSTS (/30)	FU (m)
**Abudu et al**. [5]	1999	4	34	1W, 2M, 1I	2 (50%)	1 (25%; 1D)	1 Talus loosening 1 Wound necrosis 1 Impingement	1 (25%)	19.3	57
**Lee et al**. [6]	1999	5	24	N/A	2 (40%)	1 (20%; 1D)	1 Talus collapse	0	24.2	64
**Natarajan et al**. [7]	2000	6	18	3W, 2M, 1I	1 (17%)	1 (17%; 1D)	1 Flap necrosis	2 (33%)	24.3	40
**Shekkeris et al**. [8]	2009	6	43	N/A	3 (50%)	2 (33%; 2D)	1 Talus loosening	0	21.0	116
**Ajit Singh et al**. [9]	2012	4	23	4W	2 (50%)	1 (25%; 1D)	1 Talus collapse	0	24.7	27
**Yang et al**. [10]	2017	8	33	7 W, 1M	3 (37%)	2 (25%; 1D + 1S)	1 Talus loosening	2 (25%)	20.3	113
**Kask et al**. [11]	2018	1	48	1 W	0	0	0	0	23	100
**Raciborska et al**. [12]	2021	14	14	14 W	2 (14%)	0	1 Loosening 1 Wound necrosis	N/A	22	20
**Savvidou et al**. [13]	2022	1	18	1W	0	0	0	0	-	-
**Mugla et al**. [14]	2022	10	31	10W	2 (20%)	1 (10%; 1D)	1 Persistent pain	2 (20%)	24.9	43
***Our Study***	***2024***	***2***	***56***	***2 W***	***1 (50%)***	***0***	***1 Skin necrosis***	***0***	***30***	***12***

MARGINS: W = Wide, M = Marginal, I = Intralesional

CASES W. COMPLIC. = CASES WITH COMPLICATIONS

L. R. = LOCAL RECURRENCE

(N) = Number

(y) = Years of age

(m) = Months

While the first studies relied on monoblock distal tibia endoprostheses, designed to provide the best strength and resistance to the implant [5-7, 10, 11, 13], we followed the footsteps of most recent studies who often opted for modular implants that permit a better intra-operative customization [9, 14]. Composite prostheses are generally easier to handle intra-operatively and also allow surgeons to change the length of the tibial endoprosthesis in case the tibial resection resulted in marginal or intralesional and a recut was necessary for the local control of the disease [4]. Down to the ankle, several resurfacing approaches have been proposed and used in literature to replace the native tibiotalar joint. Like others before us [5-14], we opted for fixed total ankle joint replacements to provide high stability without sacrificing the articular range of motion. Considering that in both our cases, bone tours were confined to the tibia, we chose to preserve the bone stock of the talus as much as possible. This choice was also guided by the necessity to reduce the risk of talus collapse and loosening, which had an overall incidence of over 8% in previous international literature [5-14]. None of our cases suffered from talar collapse or loosening at their latest follow-up, suggesting the effectiveness of our approach. Some authors also reported mobilizations in the tibial bone-prosthesis interface, an eventuality that did not occur in our cases, who did not show signs of proximal loosening. All previous studies described the use of intramedullary stems [5-14]. However, in one of our cases (Case 2), the implant had to be stabilized to what remained of a free vascularized fibular graft, which had replaced the native tibia in a previous surgery and whose intramedullary canal was not large enough to house a proper stem. For this reason, a custom-made lateral plate was designed to stabilize the prosthetic body with the previous vascularized fibular graft and proximally with the native tibia. This unprecedented approach was successful, as it led to good functional stability without any sign of loosening or fractures through the patient follow-up.

The same patient suffered from skin necrosis. This complication had already been described in cases treated with the distal tibia and ankle megaprostheses (global incidence in previous literature of 5%) [5-14], and the patient was prone to its occurrence since the surgical incision was made following the border of the coverage myo-cutaneous free flap used in the previous surgery. Fortunately, the area of necrosis was limited, and the combination of Closed Incisional Negative Pressure Wound Therapy with a Reverdin graft [15] effectively restored the continuity of the skin layer. Our cases did not suffer from other major postoperative complications, including infections, which had a global incidence of 15% in previous international literature.

Finally, our cases confirm that the implant of distal tibia and ankle megaprostheses, followed by a proper and accurate rehabilitation, can lead to good postoperative functional outcomes. Both our patients had a complete recovery obtaining an overall MSTS score of 30, even higher than the already good results reported in previous literature, with mean values ranged between 19 and 25. In conclusion, our outcomes suggest that megaprosthetic implants can represent a reliable alternative in cases with tumors of the distal tibia, leading to good clinical results and satisfying functional performances in a mid-term scenario.

## References

[1] Campanacci M (1999). Bone and soft tissue tumors.

[2] Hwang JS, Mehta AD, Yoon RS, Beebe KS (2014). From amputation to limb salvage reconstruction: evolution and role of the endoprosthesis in musculoskeletal oncology. J Orthop Traumatol.

[3] Zhao Z, Yan T, Guo W, Yang R, Tang X (2021). Is double-strut fibula ankle arthrodesis a reliable reconstruction for bone defect after distal tibia tumor resection? a finite element study based on promising clinical outcomes. J Orthop Surg Res.

[4] Andreani L, Ipponi E, Neri E, Capanna R (2021). 3D printing in orthopedic oncology surgery. Minerva Orthop.

[5] Abudu A, Grimer RJ, Tillman RM, Carter SR (1999). Endoprosthetic replacement of the distal tibia and ankle joint for aggressive bone tumours. Int Orthop.

[6] Lee SH, Kim HS, Park YB, Rhie TY, Lee HK (1999). Prosthetic reconstruction for tumours of the distal tibia and fibula. J Bone Joint Surg Br.

[7] Natarajan MV, Annamalai K, Williams S, Selvaraj R, Rajagopal TS (2000). Limb salvage in distal tibial osteosarcoma using a custom mega prosthesis. Int Orthop.

[8] Shekkeris AS, Hanna SA, Sewell MD, Spiegelberg BG, Aston WJ, Blunn GW, Cannon SR, Briggs TW (2009). Endoprosthetic reconstruction of the distal tibia and ankle joint after resection of primary bone tumours. J Bone Joint Surg Br.

[9] Ajit Singh V, Nasirudin N, Bernatt M (2013). Endoprosthetic reconstruction for giant cell tumors of the distal tibia: a short term review. Asia Pac J Clin Oncol.

[10] Yang P, Evans S, Khan Z, Abudu A, Jeys L, Grimer R (2017). Reconstruction of the distal tibia following resection of aggressive bone tumours using a custom-made megaprosthesis. J Orthop.

[11] Kask G, Pakarinen TK, Parkkinen J, Kuokkanen H, Nieminen J, Laitinen MK (2018). Tibia Adamantinoma Resection and Reconstruction with a Custom-Made Total Tibia Endoprosthesis: A Case Report with 8-Year Follow-Up. Case Rep Orthop.

[12] Raciborska A, Bilska K, Malesza I, Rodriguez-Galindo C, Pachuta B (2021). Distal Tibial Reconstruction in the Management of Primary Bone Tumors in Children and Adolescents. Foot Ankle Int.

[13] Savvidou OD, Gavriil P, Trikoupis I, Kaspiris A, Melissaridou DE, Papakonstantinou O, Korkolopoulou P, Papagelopoulos PJ (2022). Three-dimensional Printed Endoprosthesis for Reconstruction of the Distal Tibia and Ankle Joint After Tumor Resection. Orthopedics.

[14] Mugla W, Bauer HCF, Vogel J, Hosking KV, Campbell N, Hilton TL (2022). Modular prosthetic reconstruction for primary bone tumours of the distal tibia in ten patients. SA Orthop J.

[15] Gabriele L, Gariffo G, Grossi S, Ipponi E, Capanna R, Andrean L (2021). Closed Incisional Negative Pressure Wound Therapy (ciNPWT) in Oncological Orthopedic Surgery: Preliminary Report. Surg Technol Int.

